# Comparing a body-oriented and a cognitive online intervention for adults with depressive symptoms: A three-arm randomized controlled trial

**DOI:** 10.1016/j.invent.2026.101016

**Published:** 2026-10-21

**Authors:** Johannes Michalak, Miriam Gemander, Thomas Heidenreich, Tatiana Kvetnaya, Tobias Puntke, Martin Schultze

**Affiliations:** a Witten/Herdecke University, Department of psychology and psychotherapy, Alfred-Herrhausen-Straße 50, Witten, D – 58448, Germany; b University of Applied Science, Kanalstraße 33, Esslingen am Neckar, 73728, Germany; c Goethe-University Frankfurt, Theodor-W.-Adorno-Platz 6, Frankfurt am, D – 60323, Germany; d TaijiDao Association Münster, Hoher Heckenweg 249A, Münster, D – 48157, Germany

**Keywords:** Behavior therapy, Depression, Embodiment, Internet-based treatment

## Abstract

Based on findings from basic and clinical research indicating that the motor system can causally influence emotional processes, *Body, Breath and Mind* (BBM) was developed as a minimally guided online intervention. BBM combines body-oriented Qi Gong practice with elements of behavioral activation and aims to target embodied aspects of depression. In a pre-registered three-arm randomized controlled trial, the effects of BBM were compared with *MoodGym* (MG), an established online intervention based on cognitive behavioral therapy, and with a waitlist control group (WL). A total of 303 participants with negative and depressive mood were randomized to BBM, MG, or WL. The primary outcome was depressive symptom severity, assessed with the Patient Health Questionnaire-9 (PHQ-9). Quality of life, measured with the Short Form Health Survey (SF-36), served as a secondary outcome. Both BBM and MG showed greater reductions in somatic depressive symptoms of the PHQ-9 compared to WL. For the affective component of the PHQ-9, only MG demonstrated significantly greater reductions than WL. No significant differences between conditions were observed for changes in quality of life. The results do not provide evidence that adding body-oriented components yields specific benefits in the online treatment of depressive symptoms. However, overall, BBM showed largely comparable effects to those of MoodGym, although the findings were less consistent for affective depressive symptoms. BBM may therefore provide an additional minimally guided online intervention option for addressing negative and depressed mood, which may be of particular interest to individuals who prefer a body-oriented approach.

## Highlights

• Body, Breath & Mind (BBM) is a novel body-oriented online intervention.
• BBM reduced somatic depressive symptoms compared to waitlist control.
• No significant differences in depression outcomes were found between BBM and MoodGym
• BBM broadens online treatment options for depression.

## 1 Introduction

The interaction between the motoric system and psychological processes has attracted a lot of attention during the past decades. A large number of studies have investigated the effect of motor manipulations on affective responses. In these studies, changes in posture (e.g. stooped, straight, or control posture; Veenstra et al., 2017) or movement (e.g., slumped walking vs. walking with opposite arm and leg skipping; Peper and Lin, 2012) were experimentally induced and effects on mood and behavior responses were measured. Although the replicability of these findings has been critically discussed (e.g., Simmons and Simonsohn, 2017), recent meta-analyses have found robust effects of body manipulations on affective outcomes (Elkjær et al., 2022a; Körner et al., 2022). These analyses also accounted for publication bias and methodological differences between studies.

Building on these basic research findings from non-clinical populations, clinical research has begun to investigate the role of the motor system in affective processes involved in depression. A meta-analysis by Elkjær et al. (2022b) aggregated results from 71 studies and found consistent differences in motoric parameters between healthy participants and clinical (depression and anxiety) groups (*g* = 0.26). Specifically, they found differences in gait parameters and more overall abnormal movement (i.e., sway, posture) in individuals with depression. Moreover, several studies have investigated if manipulation of the motoric system can causally affect emotional processes involved in depression. Elkjær et al. (2022b) conducted a narrative review of these experimental studies. They found that four out of five studies reported significant effects of motor system interventions on affective outcomes.

For instance, Michalak et al. (2018) randomized depressed individuals into two conditions with different Qi Gong movements. Qi Gong, a component of traditional Chinese mind-body medicine, integrates body postures, movements, breathing techniques, and meditation (further details on Qi Gong are provided below). In one condition depressed patients practiced an upward-opening Qi Gong sequence, which contrasts with the slumped, downward posture often seen in depression (e.g., Michalak et al., 2009). In the second condition, depressed patients practiced a downward-closing Qi Gong sequence, which aligned more with the habitual posture that is characteristic for depressed individuals. The study investigated effects of these different Qi Gong movements on depressive memory characteristics. The results revealed that participants practicing the downward-closing movement displayed the negative memory bias typical for depressed individuals, recalling more negative words than positive. However, those practicing the upward-opening movement recalled a significantly higher proportion of positive words. Furthermore, the upward-opening group produced more specific autobiographical memories than the downward-closing group. This suggests that Qi Gong movements not only influence negative memory bias—commonly observed in depression (Matt et al., 1992)—but also improve the specificity of autobiographical memory, a characteristic often impaired in depression (Williams et al., 2007).

Results of studies showing that motoric manipulation can affect psychopathological processes in depression are consistent with the assumptions of the Interacting Cognitive Subsystems approach (ICS; Teasdale and Barnard, 1993). The ICS proposes that proprioceptive and kinesthetic input from the body makes a direct and important contribution to emotional information processing in depression. For instance, bodily sensations associated with a slumped posture or depressed gait might influence the implicational subsystem, which processes information in a holistic, schematic, and emotional manner, to interpret a situation as negative which in turn reinforces depressive mood and depressive body displays. This leads to a depressive interlock configuration, in which bodily and cognitive feedback loops become established that “lock” subsystems (i.e., the implicational subsystem and the body state subsystem) into a self- perpetuating configuration that maintains depression.

The results of experimental studies with healthy and clinical populations presented above and the theoretical considerations from the ICS suggest the body as a promising target for clinical interventions. In addition to the experimental evidence presented above, other research has shown that more comprehensive body-oriented programs may be effective in alleviating depression. For example, several reviews and meta-analyses have synthesized evidence on the effectiveness of Qi Gong-based therapy for depression. Guo et al. (2019) reviewed seven randomized controlled trials (RCTs) focused on Qi Gong therapy for adults with Major Depressive Disorder and reported significant effects on depressive symptoms with a moderate effect size. Liu et al. (2015) analyzed 18 RCTs involving individuals with depressive symptoms. Both meta-analyses, while acknowledging the methodological shortcomings of the included studies, suggest that Qi Gong may have positive effects on depression.

The presented evidence encouraged us to develop *Body, Breath and Mind* (BBM), an online intervention for individuals suffering from negative and depressive mood. BBM combines Qi Gong practices with value-based behavioral activation, an evidence based therapeutic approach for depression (Dimidjian et al., 2008; Jacobson et al., 2001; Martell et al., 2010). In each of the eight modules of BBM participants get video instructions for different Qi Gong practices by an experienced Qi Gong teacher. Moreover, in every module participants receive written instructions on value-based behavioral activation (e.g., instructions to clarify personally relevant values and to formulate behavioral goals in the direction of these values).

We choose Qi Gong as a core element of BBM for several reasons. Qi Gong movements can be tailored to the habitual downward-slumped posture/movement pattern that is characteristic for depressed individuals. Upward-opening exercises can support participants to learn a more upward opening posture or movement pattern, that might provide, according to the ICS, non-depressed feedback from the body state subsystem to the implicational subsystem and reduce the salience of depressive cognitive schemas. Additionally, Qi Gong movements are typically performed with mindful attention to the body. This embodied mindfulness may help participants disengage from dysfunctional ruminative thinking and reconnect with the present moment's lived experience (Farb et al., 2012; Khoury et al., 2017; Michalak et al., 2012). In addition to movement-oriented Qi Gong practices, some Qi Gong exercises focus on breathing techniques. Since there is preliminary evidence for dysfunctional breathing patterns in depression (Zamoscik et al., 2018), Qi Gong practices focused on breathing might help to functionally regulate breathing patterns in individuals prone to negative and depressed mood. Moreover, intrusive mental images are very common in depression and are often associated with intense emotions (Görgen et al., 2015; Patel et al., 2007). Rescripting these negative mental images has been shown to be effective in treating depression (Moritz et al., 2018). Since same Qi Gong practices use imagination (e.g., imagining oneself turning into a tree to improve psychological stability through ‘grounding’ and strengthening the connection to nature, Capaldi et al., 2015), Qi Gong exercises involving imagination might train participants with negative mood to form more healthy mental images. Finally, another rationale for the benefit of Qi Gong in treating negative mood is its potential ability to address a key phenomenological aspect of depression: the depletion of energy and vitality (DSM-5: criterion A5 of Major Depressive Disorder; American Psychiatric Association, 2013). Phenomenological research suggests that this loss of vitality is just as central to the experience of depression as the negative mood itself (Fuchs, 2013). The term ‘Qi’ in Qi Gong refers to ‘vital energy,’ and ‘Gong’ means ‘cultivation’ or ‘work.’ Therefore it might be speculated that Qi Gong might be especially targeted to address this embodied aspect of depression by increasing the subjective level of vitality.

In a pilot study, Michalak et al. (2024) assessed the feasibility and preliminary efficacy of BBM with 110 participants. Results showed a significant reduction in depressive symptoms with a large effect size. Participants reported high satisfaction with both the Qi Gong and behavioral activation components, as well as with the overall program. However, the dropout rate was relatively high, with 31% of participants having completed all eight modules.

Given these promising findings of the BBM pilot study, we conducted a randomized controlled trial to further evaluate its potential. To assess its absolute efficacy, BBM was compared to a waitlist control group. Additionally, we examined its relative efficacy by comparing its effects with *MoodGym* (MG, Christensen et al., 2004; Mackinnon et al., 2008), a five-module online intervention based on classical cognitive behavioral therapy principles without body-focused elements. MoodGym is one of the most widely disseminated internet-based interventions for depression in the world (Schröder et al., 2016).

In this three-arm randomized trial, we assessed the effects of the interventions on depressive symptoms and quality of life. Based on the theoretical rationale and the findings of the pilot study, we hypothesized that BBM would result in greater reductions in depressive symptoms than the waitlist control condition. We likewise expected MoodGym, as an established cognitive-behavioral intervention, to outperform the waitlist condition. In line with our preregistration, we did not expect significant differences in overall efficacy between BBM and MoodGym. This prediction reflected the expectation that differences between active psychological interventions would be relatively small and was not intended as a formal hypothesis of equivalence or non-inferiority. Given that BBM and MoodGym differ in their emphasis on body-focused elements, we additionally examined their effects on the affective and somatic dimensions of depressive symptoms and quality of life.

## 2 Methods

### 2.1 Transparency and openness

We adhered to the Journal Article Reporting Standards (Appelbaum et al., 2018) and supplementary criteria from the Consolidated Standards of Reporting Trials of Electronic and Mobile Health Applications and Online Telehealth (Eysenbach and CONSORT-EHEALTH Group, 2011). The research project was approved by the Ethics Committee of Witten/Herdecke University (16/2022) and preregistered at ClinicalTrials.gov (NCT05293691). All data and the code to reproduce the analyses are available at OSF (https://osf.io/rndx4/overview; see data and scripts). The statistical analyses were conducted by MS and TK, independent co-authors who had no involvement in the development of BBM, study design, participant recruitment, data collection, or outcome assessment.

### 2.2 Participants

Because overly rigid inclusion criteria for users testing an online intervention would limit generalizability (Fleming et al., 2018), the only inclusion criteria were legal age, sufficient language proficiency (German), and access to the internet and e-mail. The project website informed potential participants that we were looking for individuals “suffering from negative or depressive mood.” A priori exclusion criteria were psychotic and schizoaffective disorders, major depression with psychotic features, eating disorders, current substance use disorders, and acute suicidality because the minimal therapeutic support were unlikely to meet the participants' needs. Moreover, participants with psychomotor agitation in the context of a depressive disorder were excluded since the bodily activating treatment components of BBM might be not suitable for this subtype of depression. For details of participant inclusion, withdrawal, and exclusion see Fig. 1 (Consort Flow Diagram).

**Fig. 1:**
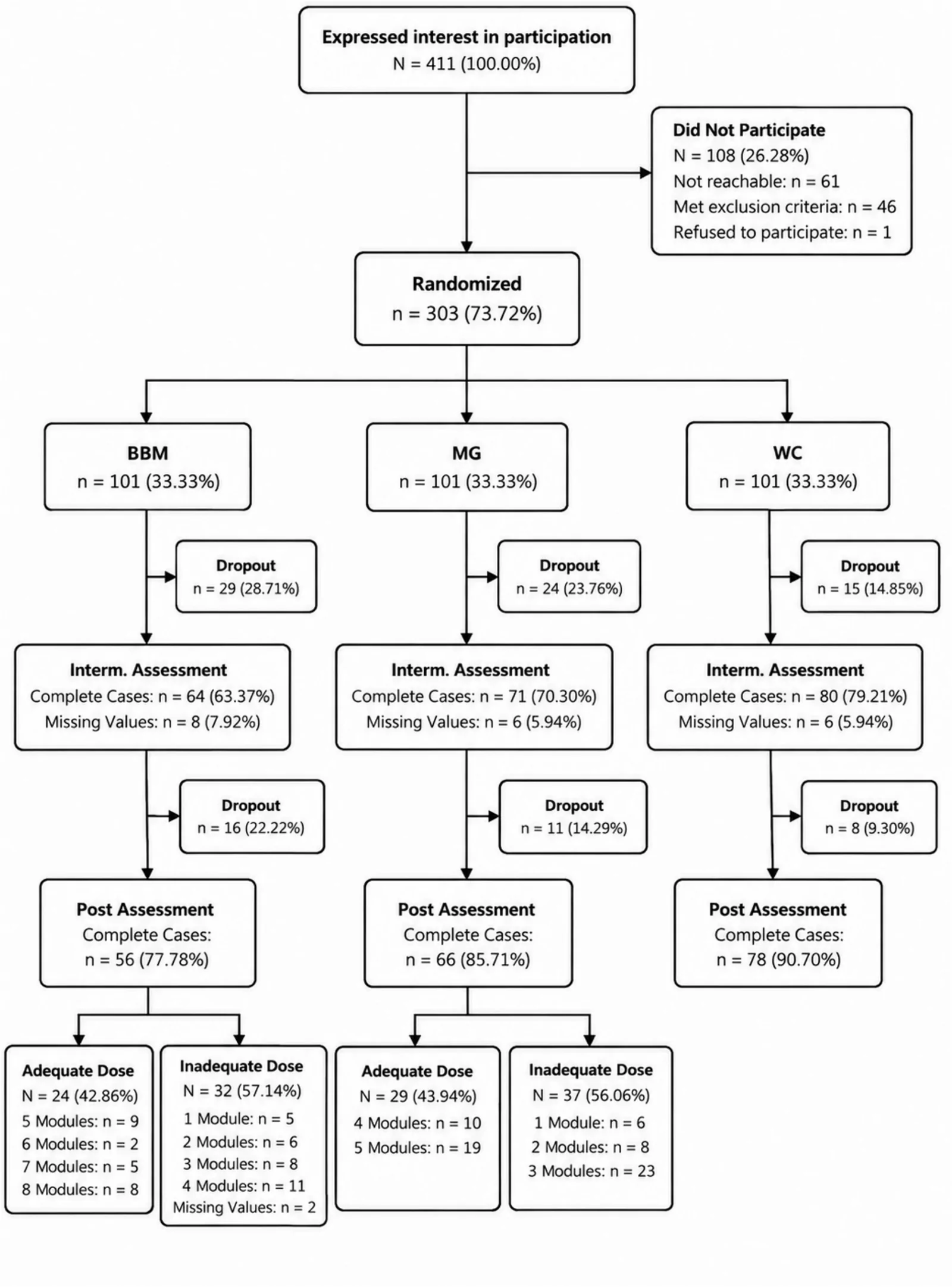
CONSORT Flow-Chart of participants (AI edited). Participants with missing values but not declared to have dropped out between baseline and intermediary assessment are those who did not participate in the intermediary assessment, but did return for the post assessment. Percentages indicate the proportion of participants progressing from the preceding node.

The target sample size of 303 individuals was determined a priori using the G*Power software with an expected effect of *d* = 0.36 (α = 0.05; β = 0.80). The expected effect was based on a study on the effectiveness of MoodGym (Löbner et al., 2018), with which the intervention was to be compared.

The final sample consisted of 303 participants with a mean age of 46.14 years (*SD* = 14.01, *Mdn* = 48). 236 participants (77.89%) identified as female, 65 (21.45%) as male, and 2 (0.66%) as non-binary. Table 1 provides an overview of baseline measures and demographic characteristics by condition.

**Table 1:** Demographic composition and baseline characteristics by condition.

Variable	BBM (*n* = 101)	MG (*n* = 101)	WC (*n* = 101)
Age *M* (*SD*)	46.29 (14.39)	47.68 (14.13)	44.45 (13.45)
Sex *n* (%)			
Male	25 (24.75%)	19 (18.81%)	21 (20.79%)
Female	75 (74.26%)	81 (80.20%)	80 (79.21%)
Non-binary	1 (0.99%)	1 (0.99%)	0 (0.00%)
Profession *n* (%)			
Student	11 (10.89%)	5 (4.95%)	14 (13.86%)
Employed	48 (47.52%)	47 (46.53%)	46 (45.54%)
Self-employed	3 (2.97%)	6 (5.94%)	7 (6.93%)
Retired	16 (15.84%)	18 (17.82%)	11 (10.89%)
Unemployed	8 (7.92%)	5 (4.95%)	11 (10.89%)
Other	15 (14.85%)	20 (19.80%)	12 (11.88%)
Highest educational level^a^*n* (%)			
Lower secondary school (Hauptschule)	2 (1.98%)	3 (2.97%)	2 (1.98%)
Intermediate Secondary School (Mittlere Reife)	9 (8.91%)	11 (10.89%)	9 (8.91%)
Abitur (University Entrance Qualification)	14 (13.86%)	13 (12.87%)	13 (12.87%)
Vocational Training (Ausbildung)	19 (18.81%)	25 (24.75%)	22 (21.78%)
University of Applied Science (Fachhochschule)	18 (17.82%)	15 (14.85%)	13 (12.87)
University (Universität)	31 (30.69%)	32 (31.68%)	39 (38.61%)
Other	8 (7.92%)	2 (1.98%)	3 (2.97%)
Psychotherapy *n* (%)			
Current	48 (47.52%)	41 (40.59%)	45 (44.55%)
Past	44 (43.56%)	49 (48.51%)	42 (41.58%)
Never	9 (8.91%)	11 (10.89%)	14 (13.86%)
Current use of psychopharmaceuticals *n* (%)	37 (36.63%)	51 (50.50%)	47 (46.53%)
Prior use of online self-help *n* (%)	11 (10.89%)	13 (12.87%)	22 (21.78%)
PHQ-9 *M* (*SD*)	21.93 (4.80)	22.32 (4.94)	20.45 (5.01)
SF-36 *M* (*SD*)			
Physical component	55.82 (20.62)	52.75 (24.37)	61.14 (24.36)
Mental component	36.99 (15.77)	38.56 (19.51)	41.68 (19.51)

*Note.* Note. BBM = Body, Breath and Mind, MG = MoodGym, WC = Waitlist control group; a. based on categories of the German education system.

Regarding mental health-related variables, 135 participants (44.55%) reported having received previous and 134 reported receiving ongoing psychotherapy (44.22%). 135 (44.55%) were currently using psychotropic medication. Additionally, 46 participants (15.18%) had prior experience with online self-help programs. At baseline, the average severity of depressive symptoms, as measured by the Patient Health Questionnaire-9 (PHQ-9), was 21.56 (*SD* = 4.96), indicating a severe level of depression severity in the sample.

### 2.3 Data collection procedures

The recruitment process was conducted through a variety of online and offline channels, including self-help groups, depression internet forums, social media, and the University of Witten/Herdecke press distribution list. Enrollment was based on self-selection. Exclusion criteria were assessed via telephone using the German version of the Structured Clinical Interview for DSM-5 (SCID, German version by Beesdo-Baum et al., 2019). The SCID interviews were conducted by graduate students in psychology who held a B.Sc. degree in psychology and had received training in SCID administration.

Informed consent, further study communication, and surveys were conducted online to maintain a low-threshold, time-efficient approach and to avoid social desirability effects or therapeutic benefits beyond the intervention that could occur in face-to-face settings (Fleming et al., 2018). Data were collected on the platform Limesurvey at three time points (pre, *peri*, post). The post-assessment was conducted eight weeks after baseline for the BBM and WC groups and five weeks after baseline for the MG group, irrespective of whether participants had completed all intervention modules. The intermediate assessment was scheduled approximately halfway through the respective intervention period, resulting in different assessment times due to the different durations of BBM and MG. To study the intervention under realistic conditions and strengthen ecological validity, no compensation or incentives were offered to the participants.

### 2.4 Random assignment and conditions

Participants were allocated to groups (BBM, MG, WC) during the ongoing recruitment process by unblinded research assistants using a pre-generated random number list in Microsoft Excel 2016 (Microsoft Corporation, 2016). Each participant was randomized immediately after eligibility had been established and was assigned consecutively according to the pre-generated randomization list. The random number list was generated by a statistician independent of the research team. Randomization was performed in blocks of six (1:1:1 ratio). Following allocation, participants received an e-mail with a link to the baseline survey. Those in the intervention and active control groups also received access to one of the two online programs. The intervention group received BBM over eight weeks; the active control group MG for five weeks. The WC received no intervention or study-related communication for eight weeks. After post-assessment (T3), WC participants were granted access to the BBM intervention.

### 2.5 Interventions

The marketing and information website stated that the study aimed to investigate two online self-help programs designed to alleviate negative and depressive mood. To minimize preference and expectation effects between the programs, both interventions were described in parallel and with equal emphasis (Rutherford et al., 2010). Following a naturalistic (pragmatic) design approach, participants were not influenced by the investigators with regard to their care as usual. During this time, they were instructed to refrain from using any self-help interventions.

*Body, Breath and Mind (BBM).* BBM was developed at the University of Witten/Herdecke and recently evaluated in a feasibility study (Michalak et al., 2024). BBM is a minimally monitored online intervention aimed to reduce depressive symptoms by combining Qi Gong elements with value-based behavioral activation (BA; Dimidjian et al., 2008; Jacobson et al., 2001; Martell et al., 2010). Each of the eight modules (duration between 60 and 80 min) begins with a Qi Gong session, including: mindful Qi Gong movement, breathing, relaxation, and imagination exercises, video guided by an advanced Qi Gong teacher. The Qi Gong exercises were selected based on embodiment research (Michalak et al., 2018, Michalak et al., 2022). Moreover, the Qi Gong exercises chosen for BBM focused on the different disharmonies supposed to be associated with depression in Traditional Chinese Medicine (for more details see Michalak et al., 2024 and the electronic supplement).

The BA module themes and exercises are presented after the Qi Gong exercises. They included psychoeducation, identification of personal values, planning and implementing value-based activities, strategies for dealing with difficulties in implementing activities, mindfulness practices, and strategies for managing rumination and preventing relapse. These elements are standard components of behavioral activation interventions (e.g., Hofheinz et al., 2017; Jacobson et al., 2001; Martell et al., 2010). The goal is to increase value-oriented actions to counteract the downward spiral of negative mood and inactivity, while fostering a reinforcement-effective behavioral structure that aligns with one's goals and values. The BA-content was mainly presented in text-based format, with audio instructions included in some modules.

Beyond practice during modules, participants were encouraged to practice Qi Gong on a daily basis (30 min) and were therefore provided with six additional videos each week. BBM was delivered on the Minddistrict platform (https://www.minddistrict.com).

*Mood Gym (MG).* MG was developed 20 years ago at the Australian National University and has been evaluated many times since (Christensen et al., 2004; Mackinnon et al., 2008; Twomey and O'Reilly, 2017). The interactive self-help program aims to prevent and reduce depressive symptoms and combines aspects of cognitive behavioral therapy with interpersonal therapy.

MG consists of an introductory session and five consecutive modules (duration per module: approximately 20–40 min). Behavioral techniques are used to change maladaptive thought and behavior patterns. The modules focus on identifying and changing distorted thoughts, increasing social and physical activity, and strengthening relaxation and problem-solving skills. They integrate psychoeducational information, animated demonstrations in the form of example characters, self-tests and interactive exercises. At the end of each module, a summary of the results is provided, allowing users to monitor their own symptom progression. Exercises for everyday life are provided to reinforce the content of the modules (a more detailed overview of MG can be found in the electronic supplement).

The German version of MG is provided free of charge by the Allgemeine Ortskrankenkasse (AOK), one of the largest public health insurance providers in Germany. Participants assigned to the Moodgym arm accessed the program via AOK's official website.

*Additional Intervention.* Participants with access to BBM or MG received minimal therapeutic support. In case of questions, technical difficulties, or serious crises, they could contact team members (graduate students in psychology, supervised by J.M.). In the event of a serious clinical crisis (e.g., emergence of suicidality or marked worsening of depressive symptoms), J.M., a licensed psychotherapist, was to be directly involved and would contact the participant to assess the situation and discuss the necessary next steps, including referral to appropriate emergency mental health services.

To boost motivation and engagement, participants received weekly emails that reminded them of the importance of regular engagement with the intervention, provided additional background information related to the respective module (e.g., references to relevant research), and encouraged participants to resume the intervention if they had not engaged with it for some time (Beatty and Binnion, 2016; Richards and Richardson, 2012; Titov et al., 2013). Furthermore, participants were free to access other mental health services and resources during the study. This reflects real-world conditions, in which online programs are often used as complementary or adjunctive interventions. Allowing such flexibility enhances the ecological validity of the study and acknowledges that some individuals may require more intensive levels of care.

### 2.6 Measures

*Primary outcome:* We assessed participants' depression symptoms with the Patient Health Questionnaire (PHQ-9, German version by Löwe et al., 2002). With 9 items, the PHQ-9 scores each of the DSM A-criteria of major depression on a scale for 0 “not at all” to 3 “nearly every day” (e.g. “I feel down, depressed, or hopeless”). Studies have shown that the PHQ-9 shows high reliability (Cronbach's α ranging from 0.86 to 0.89, test–retest reliability: *r* = 0.84) and validity (Kroenke et al., 2001) and is suitable for online use (Erbe et al., 2016). As the PHQ-9 was self-administered, the assessments were unmasked.

*Secondary outcome:* We assessed quality of life with the Short Form Health Survey (SF-36; Ware Jr and Sherbourne, 1992; German version by Morfeld et al., 2011). The SF-36 assesses mental and physical health with 36 items on eight dimensions (i.e., vitality, physical functioning, bodily pain, general health perception, role limitations due to physical health, role limitations due to emotional health, social role functioning, mental health). The SF-36 is a widely used measure of health-related quality of life with well-established reliability and validity across diverse populations (Ware Jr and Sherbourne, 1992).

### 2.7 Statistical analysis

*Primary outcome: PHQ-9.* The dimensionality of depressive symptoms has been the topic of intense debate in recent years. Recent investigations of the dimensionality of the PHQ-9 reveal either one (e.g. Bianchi et al., 2022; Gonzalez-Blanch et al., 2018) or two (e.g. Moehring et al., 2021; Ong et al., 2022) factors, with one representing affective symptoms (anhedonia, depressed mood, low self-esteem) and the other representing somatic symptoms (sleep disturbance, fatigue, appetite change, concentration problems, psychomotor impairments). Often the two factor solutions do not reflect these same concepts (e.g. Gonzalez-Blanch et al., 2018; Vu et al., 2022) and some investigations have shown the necessity for more complex dimensionality assumptions (e.g. López-Guerra et al., 2022; Tseng et al., 2024). Fried and Nesse (2015) proposed investigating depressive symptoms individually, because interventions tend to display differential effects on specific symptoms. Similarly, developments in psychological network approaches have called the common cause conceptualization of depression into question (e.g. Borsboom, 2017). In line with this approach, we analyzed the items of the PHQ-9 without imposing dimensionality assumptions by using multi-group single-indicator latent change models (McArdle and Hamagami, 2001). For comparability, we report the results of the group comparisons with respect to the sum score scale values in the Appendix. We performed the test presented by VanderWeele and Vansteelandt (2022) to check whether it is necessary to reject the common cause model entirely in this case. Additionally, we report model fit of confirmatory factor analysis (CFA) of the PHQ-9, assuming the one and two dimensional models outlined above. Sequentially more restrictive models were used to check for invariance across the three intervention groups by constraining the single-indicator latent variables to have equal variances (akin to the homoscedasticity assumption in ANOVA) and covariances (akin to measurement invariance checks in CFA). These steps were performed for the baseline measurements, the changes from baseline to the intermediary assessment, and the changes from the intermediary assessment to the post-assessment. Afterwards, mean changes were investigated by testing the total change (baseline to post) for equality across the three groups. In cases with significant inequality, we checked changes from baseline to the intermediary assessment and changes from the intermediary assessment to the post measurement. Standardized mean differences (i.e. Cohen's *d*) were determined via the standardized model results as implemented in the R-package lavaan (version 0.6–19; Rosseel, 2012). These model sequences were undertaken separately for the affective PHQ-9 items (1, 2, and 6), the somatic items (3, 4, 5, 7, and 8), and the suicidal ideation item (9). Due to the skewed distributions of the observed variables, maximum-likelihood with robust standard errors (MLR)) was used. To account for missing data, we used full information maximum-likelihood. We utilized listwise deletion analysis for the per protocol analyses described below as a sensitivity check for systematic bias introduced by handling missing this way. Missing values did not occur on the primary outcome (PHQ-9) beyond drop-out reported in Fig. 1. The aforementioned sequential models were compared using the strictly positive Satorra-Bentler χ^2^, as well as the Akaike and the Bayesian Information Criteria (AIC, BIC).

*Secondary Outcome: SF-36.* Analysis of the SF-36 closely resembled the procedure outlined above. Due to the number of items, we investigated the scale values of the eight dimensions, which were computed in line with Morfeld et al. (2005) and range from 0 (indicating bad health) to 100 (indicating good health). These scale values were analyzed in a joint multi-group single-indicator latent-change model. Again, we first checked equality of the variances and covariances across the groups, before testing global mean differences (baseline to post-measurement) and – pending a significant omnibus effect – investigating which occasions and which groups differed in their change. Missings beyond dropout occurred in 5 cases (1.6%), resulting in 22 missing values for subscales (0.7%).

*Adherence.* Adherence was assessed based on participants' reports of the most recently completed module and was defined as completion of at least 50% of the intervention modules (4 out of 8 for BBM and 3 out of 5 for MG), representing a pragmatic threshold for substantial intervention exposure. Module completion is commonly used as an indicator of adherence in digital psychological interventions, although operationalizations vary and no generally accepted threshold for adequate adherence has been established (Christensen et al., 2009; van Ballegooijen et al., 2014).

## 3 Results

### 3.1 Adherence

Fig. 1 shows the CONSORT flowchart of participants, including the categorization of adequate doses. Participation rates at post assessment differed significantly between the three groups (*χ*^2^ = 10.71, *df* = 2, *p* = .005), with post-hoc tests indicating significant differences only between WC and BBM (*χ*^2^ = 9.78, *df* = 1, *p* = .002), with a higher rate of participants completing post assessment in the waitlist control group (77.23%) than in the BBM group (55.45%).

In terms of adequate dose, BBM and MG did not differ in the proportion of participants completing at least half of the modules (BBM: 43%, MG: 44%; *χ*^2^ = 0.41, *df* = 1, *p* = .522). Analyses of depressive symptom severity and quality of life including only participants meeting this adherence criterion did not reveal a different pattern of results compared with the full-sample analyses reported below. Thus, the results of these per-protocol analyses are provided on OSF (https://osf.io/rndx4/files/osfstorage; see scripts). Dose–response associations between the number of completed modules and outcomes were not examined.

### 3.2 Reduction of depressive symptom severity

The test for the structural interpretation of the latent factor model rejected the factorial model of the PHQ-9 (*χ*^2^ = 23.27, *df* = 8, *p* = .003), indicating a necessity to investigate the items at a more detailed level. For the affective component (items 1, 2, and 6) the test did not indicate a necessity to reject the common cause approach across the three experimental groups at baseline (*χ*^2^ = 0.85, *df* = 2, *p* = .653), while it was significant for the somatic component (items 3, 4, 5, 7, and 8; *χ*^2^ = 12.15, *df* = 4, *p* = .016), again indicating a need for a more granular investigation than a single factor to differentiate effects between the three groups. A sequence of nested CFA models with all data from the first measurement occasion revealed poor model fit when assuming unidimensionality (*χ*^2^ = 84.83, *df* = 27, *p* < .001, RMSEA = 0.090, SRMR = 0.051, CFI = 0.900) or the two factor model differentiating between somatic and affective factors (*χ*^2^ = 63.93, *df* = 26, *p* < .001, RMSEA = 0.076, SRMR = 0.044, CFI = 0.932). Allowing item 9 (suicidality) to load on both factors did not remedy this misfit (*χ*^2^ = 63.93, *df* = 25, *p* < .001, RMSEA = 0.078, SRMR = 0.044, CFI = 0.931). Utilizing a multi-group CFA assuming only configural invariance between the three groups at the first occasion resulted in even worse fit (unidimensional: *χ*^2^ = 163.58, *df* = 81, *p* < .001, RMSEA = 0.107, SRMR = 0.070, CFI = 0.862, two factor: *χ*^2^ = 141.76, *df* = 78, *p* < .001, RMSEA = 0.096, SRMR = 0.065, CFI = 0.893, free item 9: *χ*^2^ = 138.38, *df* = 75, *p* < .001, RMSEA = 0.098, SRMR = 0.064, CFI = 0.894).

Sequentially constrained single-indicator latent-change models for the affective component showed the assumption of equal variances and covariances across the three groups to be tenable for baseline values and the changes between baseline and the midpoint assessment. Variances of the changes from midpoint to post differed significantly, however, so that these were allowed to vary freely in the models testing mean changes (final model: *χ*^2^ = 25.71, *df* = 24, *p* = .368). Results indicate that overall changes differed significantly between the three groups (Δ*χ*^2^ = 14.90, *df* = 6, *p* = .021). Post-Hoc tests revealed no significant differences between the three groups in changes between baseline and intermediary assessment (Δ*χ*^2^ = 11.46, *df* = 6, *p* = .075) nor in changes between intermediary and post assessment (Δ*χ*^2^ = 5.91, *df* = 6, *p* = .433), however. This indicates that the detected differences in change could not be localized to either early or late treatment stages. The overall changes (baseline to post) differed significantly only between MG and the WC (Δ*χ*^2^ = 10.11, *df* = 3, *p* = .018), while differences were not detectable when comparing BBM to either the WC (Δ*χ*^2^ = 6.60, *df* = 3, *p* = .086) or MG (Δ*χ*^2^ = 5.10, *df* = 3, *p* = .164). Table 2 shows the unconstrained mean changes for all items and reveals significant reduction of the affective component items from baseline to post in all three groups. Additionally, Table 3 depicts all pairwise comparisons of the base to post changes between the three groups.

**Table 2:** Unconstrained mean changes for all groups. *Midpoint Change* refers to the latent change variables from T1 to T2 and *Post Change* refers to latent change variables from T2 to T3 and *Overall Change* refers to their sum.

		Midpoint Change			Post Change			Overall Change		
Symptom	Group	Mean	95%-CI	Cohen's *d*	Mean	95%-CI	Cohen's *d*	Mean	95%-CI	Cohen's *d*
Anhedonia^a^	BBM	−0.31	[−0.50, −0.11]	−0.37	−0.22	[−0.42, −0.01]	−0.27	−0.52	[−0.73, −0.32]	−0.64
	MG	−0.51	[−0.70, −0.31]	−0.58	−0.05	[−0.20, 0.09]	−0.09	−0.56	[−0.75, −0.37]	−0.67
	WC	−0.18	[−0.35, −0.02]	−0.24	−0.03	[−0.20, 0.14]	−0.04	−0.21	[−0.37, −0.05]	−0.28
Depressed Mood^a^	BBM	−0.38	[−0.59, −0.18]	−0.46	0.03	[−0.19, 0.26]	0.04	−0.35	[−0.58, −0.13]	−0.42
	MG	−0.47	[−0.65, −0.29]	−0.58	−0.13	[−0.29, 0.03]	−0.19	−0.60	[−0.79, −0.40]	−0.77
	WC	−0.16	[−0.35, 0.02]	−0.18	−0.13	[−0.28, 0.02]	−0.2	−0.29	[−0.48, −0.10]	−0.38
Sleep Disturbance^b^	BBM	−0.52	[−0.80, −0.25]	−0.5	0.1	[−0.16, 0.37]	0.12	−0.42	[−0.66, −0.18]	−0.38
	MG	−0.34	[−0.54, −0.14]	−0.39	−0.25	[−0.46, −0.04]	−0.29	−0.60	[−0.84, −0.35]	−0.68
	WC	−0.02	[−0.22, 0.18]	−0.02	0.02	[−0.19, 0.23]	0.02	0.00	[−0.22, 0.23]	0.00
Fatigue^2^	BBM	−0.43	[−0.63, −0.23]	−0.52	−0.13	[−0.38, 0.13]	−0.13	−0.56	[−0.78, −0.33]	−0.65
	MG	−0.38	[−0.57, −0.18]	−0.44	−0.19	[−0.37, −0.02]	−0.28	−0.57	[−0.78, −0.35]	−0.72
	WC	−0.25	[−0.44, −0.06]	−0.28	0	[−0.16, 0.15]	0	−0.25	[−0.43, −0.07]	−0.28
Appetite Change^b^	BBM	−0.33	[−0.56, −0.10]	−0.36	0.01	[−0.26, 0.28]	0.01	−0.31	[−0.53, −0.09]	−0.34
	MG	−0.34	[−0.56, −0.12]	−0.34	0.12	[−0.10, 0.34]	0.13	−0.23	[−0.42, −0.04]	−0.22
	WC	0.01	[−0.19, 0.22]	0.01	−0.08	[−0.27, 0.11]	−0.09	−0.07	[−0.27, 0.14]	−0.08
Low Self-Esteem^a^	BBM	−0.38	[−0.57, −0.20]	−0.48	−0.19	[−0.38, 0.01]	−0.27	−0.57	[−0.80, −0.35]	−0.74
	MG	−0.33	[−0.53, −0.14]	−0.39	−0.14	[−0.32, 0.04]	−0.18	−0.47	[−0.67, −0.28]	−0.57
	WC	−0.28	[−0.48, −0.07]	−0.28	−0.17	[−0.33, −0.01]	−0.24	−0.45	[−0.62, −0.28]	−0.52
Concentration Problems^b^	BBM	−0.23	[−0.47, 0.00]	−0.24	−0.17	[−0.40, 0.06]	−0.21	−0.40	[−0.61, −0.20]	−0.45
	MG	−0.16	[−0.35, 0.03]	−0.18	−0.15	[−0.32, 0.02]	−0.22	−0.30	[−0.54, −0.07]	−0.40
	WC	−0.2	[−0.37, −0.03]	−0.25	0.04	[−0.12, 0.21]	0.06	−0.15	[−0.33, 0.02]	−0.19
Psychomotor Impairment^b^	BBM	−0.05	[−0.24, 0.13]	−0.07	−0.18	[−0.36, −0.00]	−0.26	−0.23	[−0.40, −0.06]	−0.33
	MG	−0.19	[−0.38, −0.00]	−0.22	−0.07	[−0.29, 0.14]	−0.08	−0.26	[−0.47, −0.06]	−0.30
	WC	−0.04	[−0.20, 0.12]	−0.06	0.07	[−0.10, 0.24]	0.08	0.03	[−0.14, 0.19]	0.03
Suicidality	BBM	0.03	[−0.10, 0.16]	0.05	−0.02	[−0.14, 0.11]	−0.04	0.01	[−0.11, 0.13]	0.01
	MG	−0.00	[−0.11, 0.11]	−0.00	−0.04	[−0.19, 0.11]	−0.07	−0.04	[−0.19, 0.12]	−0.07
	WC	0.13	[−0.15, 0.27]	0.23	−0.08	[−0.19, 0.04]	−0.15	0.05	[−0.08, 0.18]	0.08

*Note. Note.* BBM = Body, Breath and Mind, MG = MoodGym, WC = Waitlist control;group.

a Item related to the affective component.

b Item related to the somatic component.

**Table 3:** Pairwise group mean comparisons of all overall changes (baseline to post) for the PHQ-9.

	BBM vs. MG			BBM vs. WC			MG vs. WC		
Symptom	Est.	95%-CI	Cohen's *d*	Est.	95%-CI	Cohen's *d*	Est.	95%-CI	Cohen's *d*
Anhedonia^1^	0.04	[−0.24, 0.31]	0.07	−0.31	[−0.57. -0.06]	−0.38	−0.35	[−0.60, −0.11]	−0.45
Depressed Mood^1^	0.24	[−0.06, 0.54]	0.33	−0.06	[−0.36, 0.23]	−0.03	−0.31	[−0.58, −0.04]	−0.35
Sleep Disturbance^2^	0.16	[−0.18, 0.50]	0.19	−0.42	[−0.75, −0.10]	−0.44	−0.58	[−0.91, −0.25]	−0.63
Fatigue^2^	−0.01	[−0.33, 0.31]	−0.01	−0.31	[−0.60, −0.02]	−0.38	−0.30	[−0.59, −0.02]	−0.38
Appetite Change^2^	−0.11	[−0.40, 0.18]	−0.13	−0.27	[−0.57, 0.03]	−0.28	−0.16	[−0.44, 0.12]	−0.15
Low Self-Esteem^1^	−0.11	[−0.40, 0.19]	−0.14	−0.13	[−0.41, 0.16]	−0.14	−0.02	[−0.28, 0.24]	0.00
Concentration Problems^2^	−0.10	[−0.42, 0.21]	−0.13	−0.26	[−0.53, 0.02]	−0.35	−0.15	[−0.45, 0.14]	−0.21
Psychomotor Impairment^2^	0.04	[−0.23, 0.31]	0.05	−0.25	[−0.49, −0.01]	−0.31	−0.29	[−0.56, −0.02]	−0.36
Suicidality	0.05	[−0.15, 0.24]	0.09	−0.04	[−0.22, 0.14]	−0.07	−0.09	[−0.29, 0.12]	−0.15

*Note.* BBM = Body, Breath and Mind, MG = MoodGym, WC = Waitlist control group.

In regards to the somatic depression symptoms, equality across the three groups was sustained for all variances and covariances (final model: *χ*^2^ = 98.66, *df* = 90, *p* = .250). The omnibus test of equal changes revealed significant mean differences in total change (Δ*χ*^2^ = 18.63, *df* = 10, *p* = .045). As was the case in the previous section, these changes were neither directly attributable to changes between baseline and the midpoint (Δ*χ*^2^ = 16.99, *df* = 10, *p* = .075) nor those between the midpoint and post (Δ*χ*^2^ = 15.15, *df* = 10, *p* = .127). Comparing the overall changes between the three groups revealed significant differences between both intervention groups and the WC (BBM: Δ*χ*^2^ = 11.60, *df* = 5, *p* = .041; MG: Δ*χ*^2^ = 12.56, *df* = 5, *p* = .028), while the two did not differ significantly (Δ*χ*^2^ = 2.60, *df* = 5, *p* = .761). Again, Table 2 shows the details of the unconstrained means of the latent change variables. Direct comparisons of the baseline to post are shown in Table 3 and indicate local differences between the intervention groups and the WC for sleep disturbance, fatigue, and psychomotor impairment.

Analysis of the suicidality item indicated equality of variances across all three groups for the baseline and both change variables (final model: *χ*^2^ = 6.99, *df* = 6, *p* = .322). The test for overall change equality between the three groups revealed no significant differences (Δ*χ*^2^ = 0.68, *df* = 2, *p* = .711). Detailed mean inspections (see Table 2) show that none of the mean changes was significantly different from zero.

The originally preregistered analyses of the PHQ-9 sum score are presented in the Appendix. It showed greater reductions for both BBM (mean difference = −2.27, Cohen's *d* = −0.54) and MG (mean difference = −2.34, *d* = −0.54) compared with WL, whereas BBM and MG did not differ (mean difference = 0.07, *d* = 0.00).

### 3.3 Changes in quality of life

Sequentially constrained models indicated no evidence for group differences in variances or covariances at baseline. Model comparisons suggested that the covariance structure of changes from baseline to the intermediate assessment differed between groups, whereas the covariance structure of changes from the intermediate to the post-assessment did not differ significantly. The final model showed an acceptable fit, χ^2^(160) = 182.30, *p* = .109.

Effect size estimates for mean changes from baseline to post-assessment in the SF-36 dimensions are shown in Fig. 2. Descriptively, BBM and MG showed small to moderate improvements across most SF-36 subscales, whereas effects in the WC were generally smaller. However, constraining the groups to equal overall baseline-to-post changes did not significantly worsen model fit, Δχ^2^(16) = 13.60, *p* = .628. Thus, although the descriptive pattern suggested somewhat larger improvements in BBM and MG, the model comparison did not provide evidence for statistically significant group differences in overall change across the eight SF-36 subscales.

**Fig. 2:**
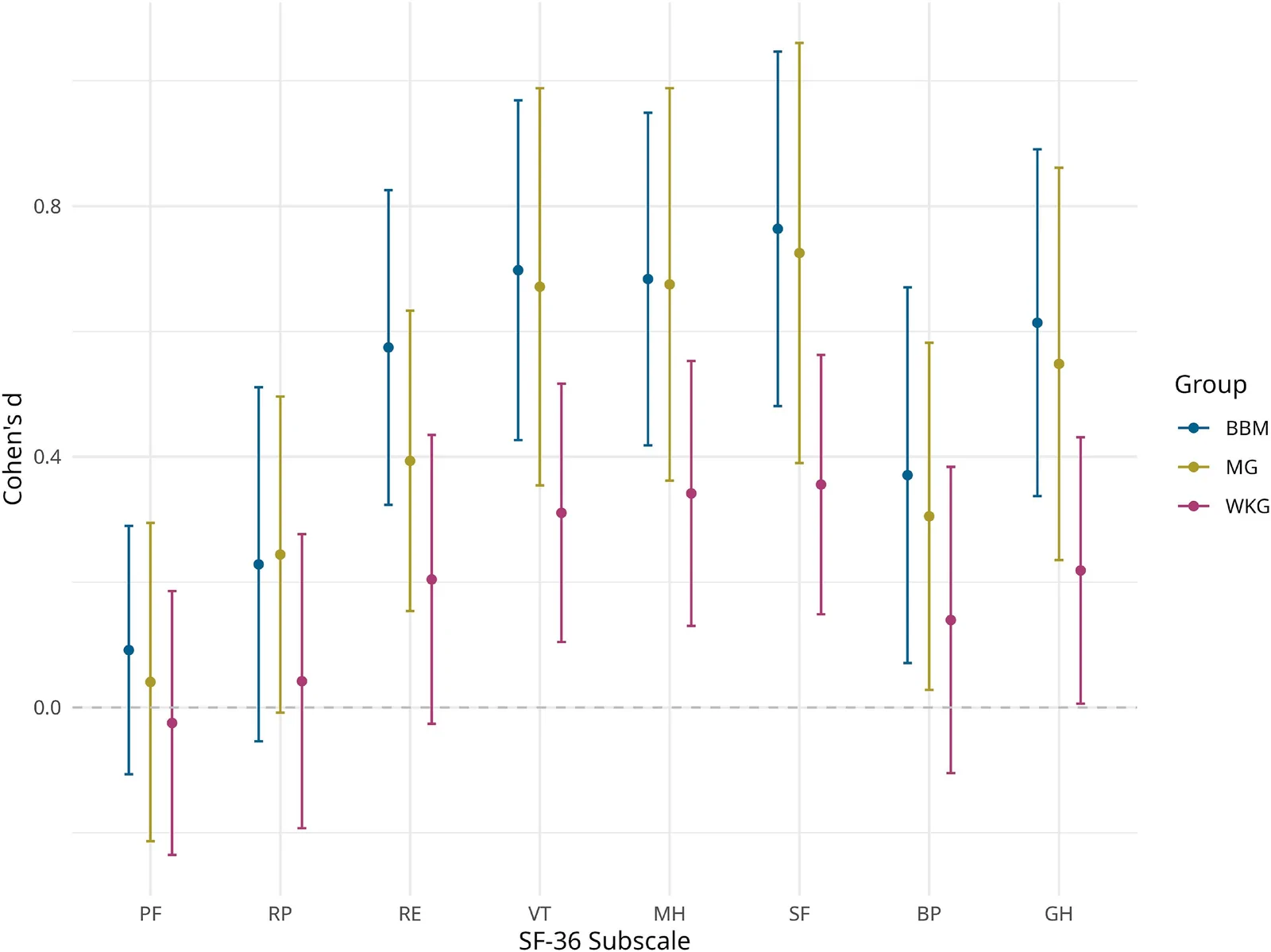
Effect size estimates (Cohen's d) of mean baseline to post changes (and their 95% confidence intervals) of all SF-36 subscales for the three groups. *Note.* PF: Physical functioning, RP: Role limitations due to physical problems, RE: Role limitations due to emotional problems, VT: Vitality, MH: Mental Well-Being, SF: Social Functioning, BP: Bodily Pain, GH: General Health.

### 3.4 Adverse events

Adverse events were not systematically assessed. However, no adverse events (AEs) or serious adverse events (SAEs) were reported during the intervention period, and no serious clinical crisis requiring direct involvement of the licensed psychotherapist occurred. Clinically significant deterioration (≥6-point increase on the PHQ-9) was observed in 0/56 (0%) participants in the BBM condition, 1/66 (1.5%) in the MoodGym condition, and 2/78 (2.6%) in the waitlist condition.

## 4 Discussion

The present study investigated the efficacy of BBM, an online intervention that combines body-oriented Qi Gong exercises with behavioral activation, in a three-arm randomized controlled trial. The effects of BBM were compared with MoodGym, a widely used online intervention for depressed mood, and with a waitlist control group. Both BBM and MoodGym showed significantly greater reductions in somatic depressive symptoms (i.e., sleep disturbances, fatigue, and psychomotor impairments) than the waitlist control group. The effects size of this reduction corresponds to those reported in the literature on internet intervention with minimal support (Plessen et al., 2025). However, with regard to the affective component of the PHQ-9, only MoodGym outperformed the waitlist control group, whereas participants in the BBM condition did not show significantly greater improvements than those in the waitlist group.

Several explanations for the absence of significant effects of BBM on affective symptoms should be considered. First, BBM may be better suited to individuals with less severe or subclinical depressive symptoms. Second, although the Qi Gong exercises were specifically selected to target embodied processes associated with depression, it remains unclear whether the intended mechanisms, such as changes in posture and movement patterns, embodied mindfulness, or subjective vitality, were sufficiently activated through self-guided online practice. Finally, the behavioral activation component of BBM was delivered predominantly in text-based form, which may have provided less support for translating therapeutic principles into behavioral change than more interactive or therapist-supported formats. Future studies should directly assess these potential mechanisms and examine for whom and under which delivery conditions the different components of BBM are most effective.

Examining pre–post changes, significant improvements were observed across all PHQ-9 items except suicidality in both the BBM and MoodGym conditions, with predominantly moderate effect sizes. A closer inspection of the pre–post changes at the item level may help to contextualize why MoodGym, but not BBM, showed a significant advantage over the waitlist condition for the affective symptom component. Descriptively, this pattern appears to be primarily driven by the depressed mood item, whereas changes in the other two affective items were similar across BBM and MoodGym. Importantly, however, the mean changes did not differ significantly between BBM and MoodGym. Consistent with prior research on digital psychological interventions (Tong et al., 2023), participants in the waitlist condition also showed significant reductions in six PHQ-9 items, albeit with small effect sizes.

In interpreting our findings, it should be noted that MoodGym comprised five modules of approximately 20–40 min each, whereas BBM comprised eight modules of approximately 60–80 min each. Thus, the interventions differed substantially in the amount of intervention content offered to participants. The finding that both interventions produced broadly comparable effects despite this difference raises important questions regarding intervention efficiency and active mechanisms. However, the present study was not designed to determine whether a shorter version of BBM would have produced similar effects or whether the additional content and time required by BBM contributed to outcomes not adequately captured by the measures used in the present study. Accordingly, the comparable effects should not be interpreted as demonstrating greater efficiency of MoodGym. Future studies could systematically vary intervention dosage and examine mediating processes to identify which components and levels of treatment intensity are necessary to achieve beneficial effects.

The BBM effect sizes observed in the present study were smaller than those reported in the BBM pilot study (Michalak et al., 2024). These differences may be attributable, at least in part, to methodological differences between the studies. For example, in contrast to the pilot study, many participants in the present RCT were recruited through self-help groups, which may have resulted in a higher proportion of individuals with persistent or treatment-resistant depression in the present sample. This possibility is also consistent with the high baseline symptom severity and the substantial proportion of participants receiving concurrent psychotherapy or psychotropic medication. For individuals with severe or persistent depressive symptoms, a minimally guided intervention such as BBM may not provide sufficient support as a stand-alone intervention. Moreover, future research should examine whether treatment intensity or the degree of professional guidance should be adapted to participants' symptom severity and treatment needs.

The effect sizes for BBM and MG on quality of life, our secondary outcome, tended to be descriptively larger than those observed in the WC group. However, the omnibus model comparison did not provide evidence for statistically reliable between-group differences across the SF-36 scales. This finding suggests that the descriptive pattern should be interpreted cautiously. The absence of significant group differences in quality of life despite improvements in depressive symptoms may have several explanations. Changes in broader aspects of functioning and quality of life may develop more slowly than changes in depressive symptoms and may therefore not yet have been detectable at the post-intervention assessment. Moreover, the SF-36 assesses broad domains of health-related quality of life and may be less sensitive to short-term intervention-related changes than a symptom-specific measure such as the PHQ-9.

Although adverse events were not systematically assessed, no AEs or SAEs were reported, and clinically significant deterioration was rare across all three conditions, providing no indication of an increased risk of negative effects associated with BBM. With regard to adherence, no significant differences were found between BBM and MoodGym in terms of completing an adequate dose of the intervention. This suggests that, within the BBM intervention, the inclusion of body-oriented exercises, which may require activation and effort and thus be challenging for some individuals with depression, did not result in lower adherence compared with MoodGym. At the same time, the relatively low rates of participants completing an adequate intervention dose in both conditions indicate that treatment adherence remains a substantial challenge in minimally guided digital interventions, which is consistent with previous research (Eysenbach, 2005; Beatty and Binnion, 2016). The higher rate of post-assessment completion in the waitlist control group compared with the BBM group should be considered separately from treatment adherence. This difference may be explained by the fact that completion of the post-assessment was a prerequisite for gaining access to the BBM intervention, thereby providing a strong incentive for participants in the waitlist condition to complete the assessment.

Taken together, although experimental findings in both healthy and clinical populations suggest that bodily manipulations can influence emotional processes and may contribute to the maintenance of depressive disorders, our results do not support the assumption that adding body-oriented components yields specific benefits in the online treatment of depressive symptoms. This aligns with broader evidence indicating negligible differences between treatment modalities in digital interventions for depression (Plessen et al., 2025).

Based on the present findings, two directions for future research appear particularly relevant. A first priority is the person-centered refinement of BBM. The relatively demanding Qi Gong shaking exercise introduced in Module 1 and the overall module length of 60–80 min may have been taxing for participants with severe depressive symptoms. Following a person-centered approach to intervention development (Yardley et al., 2015), qualitative research with individuals from the target population could identify barriers to engagement and inform decisions about intervention length, sequencing, and the selection of body-oriented exercises.

A second direction is to examine whether other forms of body-based exercise may be more effective in augmenting psychological components of digital interventions. Evidence from non-digital interventions suggests beneficial effects of exercise programs on depressive symptoms (Wolf et al., 2024) and potential benefits of aerobic exercise as an adjunct to psychological interventions (Bryant et al., 2023). Future factorial or component-based trials could therefore directly compare different body-oriented components, such as Qi Gong and aerobic exercise, when added to otherwise comparable digital psychological interventions. Such designs would allow a more direct test of whether and which body-oriented components provide incremental benefits.

Several limitations should be considered when interpreting the findings of our study. First, the sample was self-selected and recruited primarily through online channels, which may limit generalizability. In terms of representativeness, the sample showed some typical deviations from the general population, which are commonly observed in studies investigating internet-based mental health interventions (Crisp and Griffiths, 2014; Batterham and Calear, 2017). For example, the proportion of female participants was higher than in the general population, which is consistent with evidence showing that women are more likely to seek help for mental health problems (Mackenzie et al., 2006) and are disproportionately affected by depression compared to men. Moreover, participants tended to have higher educational qualifications, which may be related to a higher level of health literacy and to greater openness to participate in research (Donkin et al., 2012). Although such deviations should be discussed as a potential source of sampling bias, a study by Donkin et al. (2012) found no evidence that self-selection significantly biased outcomes or limited generalizability to public or clinical settings in an internet trial for depression.

Adherence was assessed based on participants' self-reported module completion rather than objective platform usage data because comparable log data were not available for both interventions; in particular, the research team had no access to usage metrics for MoodGym, which was accessed through the AOK website. Thus, self-reported module completion may have been subject to recall or reporting bias.

Following the typology proposed by Goldberg et al. (2023), the waitlist condition should not be considered an inert control, as participants were allowed to continue ongoing psychotherapy, psychotropic medication, and other health services. Thus, the comparison with BBM reflects the added effect of providing BBM against a background of heterogeneous treatment as usual. In contrast, the comparison between BBM and MoodGym represents a comparative efficacy comparison between two digital interventions. Because these interventions differed in both content and intensity, this comparison allows conclusions about their relative overall effects but does not isolate the effects of specific intervention components or intervention intensity.

Because the interventions differed in duration, the intermediate assessments were scheduled to occur approximately halfway through each intervention rather than at identical time points. Consequently, differences in changes involving the intermediate assessment may partly reflect differences in elapsed time between assessments. Another limitation is that it is not possible to determine the relative contribution of the Qi Gong and the behavior activation elements and their compatibility in BBM (O'Toole et al., 2025). Finally, additional contacts and the initiation of other treatments during the intervention period were not systematically assessed and therefore cannot be ruled out as factors contributing to changes observed during the study.

## 5 Conclusions

To conclude, our study found that BBM showed largely comparable effects to those of MoodGym, although the findings were less consistent for affective depressive symptoms. Taken together, the findings suggest that BBM may provide an additional minimally guided online intervention option, which may be of particular interest to individuals who prefer a body-oriented approach.

## Clinical trials

The results of this clinical trial and any associated work have been posted in a registry.

This clinical trial was registered with number NCT05293691 (ClinicalTrials.gov).

## Informed consent and patient details

Written informed consent to take part in the study and to publish the article has been obtained from all participants or their legal representatives. The privacy rights of participants have been observed.

## Studies in human

This study was performed in compliance with relevant laws, regulatory frameworks and guidelines where the research took place.

This study was approved by the Ethics Committee of Witten/Herdecke University.

(Approval No. 16/2022)

## Funding

No external funding was received for this study.

## Declaration of competing interest

The authors declare that they have no known competing financial interests or personal relationships that could have appeared to influence the work reported in this paper.

## Figures and Tables

**Table A1:** Unconstrained mean changes of the PHQ-9 sum scores for all groups. Midpoint Change refers to the latent change variables from T1 to T2 and Post Change refers to latent change variables from T2 to T3 and Overall Change refers to their sum.

	Midpoint Change			Post Change			Overall Change		
Group	Mean	95%-CI	Cohen's *d*	Mean	95%-CI	Cohen's *d*	Mean	95%-CI	Cohen's *d*
BBM	−2.48	[−3.66, −1.31]	−0.54	−1.10	[−2.24, 0.05]	−0.31	−3.58	[−4.70, −2.46]	−0.84
MG	−2.71	[−3.71, −1.70]	−0.59	−0.94	[−1.82, −0.06]	−0.26	−3.64	[−4.80, −2.49]	−0.85
WC	−0.92	[−1.90, 0.06]	−0.20	−0.38	[−1.13, 0.37]	−0.11	−1.31	[−2.30, −0.31]	−0.31

*Note.* Note. BBM = Body, Breath and Mind, MG = MoodGym, WC = Waitlist control group

## Appendix A PHQ-9 Sum score analysis

To allow for comparisons with other studies intervention studies investigating the PHQ-9 as the primary outcome, we report the results regarding sum scores in this Appendix. Aligning with the procedure reported for the item-level analyses, we used multi-group single-indicator latent change models (McArdle and Hamagami, 2001) to determine effects. Akin to the assumptions of homoscedasticity imposed in ANOVA, we checked variance equality of the change scores across the three groups using model comparisons of constrained and unconstrained models. The assumption was deemed adequate for all three variables (baseline: Δχ^2^ = 0.26, df = 2, *p* = .880, baseline-to-intermediary: Δχ^2^ = 0.24, df = 2, *p* = .887, intermediary-to-post: Δχ^2^ = 1.18, df = 2, *p* = .555) and was retained for mean comparisons. A global difference in changes between the three groups could be detected (Δχ^2^ = 11.35, df = 2, *p* = .003). Post-hoc tests revealed differences in baseline-to-post changes between BBM and WC (Δχ^2^ = 9.24, df = 1, *p* = .002) and between MG and WC (Δχ^2^ = 7.61, df = 1, *p* = .006), while BBM and MG did not differ significantly (Δχ^2^ = 0.01, df = 1, *p* = .936). Table A.1 shows the unconstrained mean changes of the PHQ-9 sum scores. Pairwise comparisons of the overall changes reveal significant differences between both intervention groups and the control (BBM: Estimate = -2.27, 95% CI: [-3.77, -0.78], Cohen's d = -0.54, MG: Estimate = -2.34, 95% CI: [-3.86, -0.82], Cohen's d = -0.54), while the two did not differ from each other (Estimate = 0.07, 95% CI: [-1.55, 1.68], Cohen's d = 0.00).

## References

[bb0005] American Psychiatric Association (2013).

[bb0010] Appelbaum M., Cooper H., Kline R.B., Mayo-Wilson E., Nezu A.M., Rao S.M. (2018). Journal article reporting standards for quantitative research in psychology: the APA Publications and Communications Board task force report. Am. Psychol..

[bb0020] Batterham P.J., Calear A.L. (2017). Preferences for internet-based mental health interventions in an adult online sample: findings from an online community survey. JMIR Ment. Health.

[bb0025] Beatty L., Binnion C. (2016). A systematic review of predictors of, and reasons for, adherence to online psychological interventions. Int. J. Behav. Med..

[bb0030] Beesdo-Baum K., Zaudig M., Wittchen H.-U. (2019).

[bb0035] Bianchi R., Verkuilen J., Toker S., Schonfeld I.S., Gerber M., Brähler E., Kroenke K. (2022). Is the PHQ-9 a unidimensional measure of depression? A 58,272-participant study. Psychol. Assess..

[bb0040] Borsboom D. (2017). A network theory of mental disorders. World Psychiatry.

[bb0045] Bryant R.A., Dawson K.S., Azevedo S., Yadav S., Cahill C., Kenny L., Maccallum F., Tran J., Rawson N., Tockar J., Garber B., Keyan D. (2023). Augmenting trauma-focused psychotherapy for post-traumatic stress disorder with brief aerobic exercise in Australia: a randomised clinical trial. Lancet Psychiatry.

[bb0050] Capaldi C., Passmore H.-A., Nisbet E., Zelenski J., Dopko R. (2015). Flourishing in nature: a review of the benefits of connecting with nature and its application as a wellbeing intervention. Int. J. Wellbeing.

[bb0060] Christensen H., Griffiths K.M., Farrer L. (2009). Adherence in internet interventions for anxiety and depression: systematic review. J. Med. Internet Res..

[bb0055] Christensen H., Griffiths K.M., Jorm A.F. (2004). Delivering interventions for depression by using the internet: randomised controlled trial. BMJ.

[bb0065] Crisp D.A., Griffiths K.M. (2014). Participating in online mental health interventions: who is most likely to sign up and why?. Depress. Res. Treat..

[bb0070] Dimidjian S., Martell C.R., Addis M.E., Herman-Dunn R., Barlow D.H. (2008). Clinical Handbook of Psychological Disorders: A Step-by-step Treatment Manual.

[bb0075] Donkin L., Hickie I.B., Christensen H., Naismith S.L., Neal B., Cockayne N.L., Glozier N. (2012). Sampling bias in an internet treatment trial for depression. Transl. Psychiatry.

[bb0080] Elkjær E., Mikkelsen M.B., Michalak J., Mennin D.S., O'Toole M.S. (2022). Expansive and contractive postures and movement: a systematic review and meta-analysis of the effect of motor displays on affective and behavioral responses. Perspect. Psychol. Sci..

[bb0085] Elkjær E., Mikkelsen M.B., Michalak J., Mennin D.S., O'Toole M.S. (2022). Motor alterations in depression and anxiety disorders: a systematic review and meta-analysis. J. Affect. Disord..

[bb0090] Erbe D., Eichert H.-C., Rietz C., Ebert D.D. (2016). Interformat reliability of the Patient Health Questionnaire: validation of the computerized version of the PHQ-9. Internet Interv..

[bb0095] Eysenbach G. (2005). The law of attrition. J. Med. Internet Res..

[bb0100] Eysenbach G., CONSORT-EHEALTH Group (2011). CONSORT-EHEALTH: improving and standardizing evaluation reports of web-based and mobile health interventions. J. Med. Internet Res..

[bb0105] Farb N.A.S., Anderson A.K., Segal Z.V. (2012). The mindful brain and emotion regulation in mood disorders. Can. J. Psychiatr..

[bb0110] Fleming T., Bavin L., Lucassen M., Stasiak K., Hopkins S., Merry S. (2018). Beyond the trial: systematic review of real-world uptake and engagement with digital self-help interventions for depression, low mood, or anxiety. J. Med. Internet Res..

[bb0115] Fried E.I., Nesse R.M. (2015). Depression sum-scores don't add up: why analyzing specific depression symptoms is essential. BMC Med..

[bb0120] Fuchs T. (2013). Depression, intercorporeality and interaffectivity. J. Conscious. Stud..

[bb2120] Goldberg Simon B., Sun Shufang, Carlbring Per, Torous John (2023). Selecting and describing control conditions in mobile health randomized controlled trials: a proposed typology. npj Digital Medicine.

[bb0125] González-Blanch C., Medrano L.A., Muñoz-Navarro R., Ruíz-Rodríguez P., Moriana J.A., Limonero J.T., Schmitz F., Cano-Vindel A., on behalf of the PsicAP Research Group (2018). Factor structure and measurement invariance across various demographic groups and over time for the PHQ-9 in primary care patients in Spain. PLoS One.

[bb0130] Görgen S.M., Joormann J., Hiller W., Witthöft M. (2015). The role of mental imagery in depression: negative mental imagery induces strong implicit and explicit affect in depression. Front. Psychol..

[bb0135] Guo L., Kong Z., Zhang Y. (2019). Qigong-based therapy for treating adults with major depressive disorder: a meta-analysis of randomized controlled trials. Int. J. Environ. Res. Public Health.

[bb0140] Hofheinz C., Heidenreich T., Michalak J. (2017).

[bb0145] Jacobson N.S., Martell C.R., Dimidjian S. (2001). Behavioral activation treatment for depression: returning to contextual roots. Clin. Psychol. Sci. Pract..

[bb0150] Khoury B., Knäuper B., Pagnini F., Trent N., Chiesa A., Carrière K. (2017). Embodied mindfulness. Mindfulness.

[bb0155] Körner R., Röseler L., Schütz A., Bushman B.J. (2022). Dominance and prestige: Meta-analytic review of experimentally induced body position effects on behavioral, self-report, and physiological dependent variables. Psychol. Bull..

[bb0160] Kroenke K., Spitzer R.L., Williams J.B.W. (2001). The PHQ-9: validity of a brief depression severity measure. J. Gen. Intern. Med..

[bb0165] Liu X., Clark J., Siskind D., Williams G.M., Byrne G., Yang J.L., Doi S.A. (2015). A systematic review and meta-analysis of the effects of qigong and tai chi for depressive symptoms. Complement. Ther. Med..

[bb0170] Löbner M., Pabst A., Stein J., Dorow M., Matschinger H., Luppa M., Maroß A., Kersting A., König H.-H., Riedel-Heller S.G. (2018). Computerized cognitive behavior therapy for patients with mild to moderately severe depression in primary care: a pragmatic cluster randomized controlled trial (@ktiv). J. Affect. Disord..

[bb0175] López-Guerra V.M., López-Núñez C., Vaca-Gallegos S.L., Torres-Carrión P.V. (2022). Psychometric properties and factor structure of the Patient Health Questionnaire-9 as a screening tool for depression among Ecuadorian college students. Front. Psychol..

[bb0180] Löwe B., Spitzer R.L., Zipfel S., Herzog W. (2002).

[bb0185] Mackenzie C.S., Gekoski W.L., Knox V.J. (2006). Age, gender, and the underutilization of mental health services: the influence of help-seeking attitudes. Aging Ment. Health.

[bb0190] Mackinnon A., Griffiths K.M., Christensen H. (2008). Comparative randomised trial of online cognitive–behavioural therapy and an information website for depression: 12-month outcomes. Br. J. Psychiatry.

[bb0195] Martell C.R., Dimidjian S., Herman-Dunn R. (2010).

[bb0200] Matt G.E., Vázquez C., Campbell W.K. (1992). Mood-congruent recall of affectively toned stimuli: a meta-analytic review. Clin. Psychol. Rev..

[bb0205] McArdle J.J., Hamagami F., Collins L.M., Sayer A.G. (2001). New Methods for the Analysis of Change.

[bb0225] Michalak J., Aranmolate L., Bonn A., Grandin K., Schleip R., Schmiedtke J., Quassowsky S., Teismann T. (2022). Myofascial tissue and depression. Cogn. Ther. Res..

[bb0215] Michalak J., Burg J.M., Heidenreich T. (2012). Don't forget your body: mindfulness, embodiment, and the treatment of depression. Mindfulness.

[bb0220] Michalak J., Chatinyan A., Chourib H., Teismann T. (2018). The impact of upward versus downward movement patterns on memory characteristics of depressed individuals. Psychopathology.

[bb0230] Michalak J., Puntke T., Trimpop L., Bohn A., Schell E., Reichert H., Senker K., Heidenreich T. (2024). Body, breath, and mind: feasibility of an internet intervention for depressive symptoms combining Qi Gong and behavioral activation. Cogn. Ther. Res..

[bb0210] Michalak J., Troje N.F., Fischer J., Vollmar P., Heidenreich T., Schulte D. (2009). Embodiment of sadness and depression—gait patterns associated with dysphoric mood. Psychosom. Med..

[bb0235] Microsoft Corporation (2016).

[bb0240] Moehring A., Guertler D., Krause K., Bischof G., Rumpf H.-J., Batra A., Wurm S., John U., Meyer C. (2021). Longitudinal measurement invariance of the patient health questionnaire in a German sample. BMC Psych..

[bb0245] Morfeld M., Bullinger M., Nantke J., Brähler E. (2005). Die Version 2.0 des SF-36 Health Survey—Ergebnisse einer bevölkerungsrepräsentativen Studie. Soz. Praventivmed..

[bb0250] Morfeld M., Kirchberger I., Bullinger M. (2011).

[bb0255] Moritz S., Ahlf-Schumacher J., Hottenrott B., Peter U., Franck S., Schnell T., Peter H., Schneider B.C., Jelinek L. (2018). We cannot change the past, but we can change its meaning. A randomized controlled trial on the effects of self-help imagery rescripting on depression. Behav. Res. Ther..

[bb0265] O'Toole M.S., Mikkelsen M.B., Arch J.J., Tauber N.M., Elkjær E., Michalak J. (2025). Compatibility of components in cognitive behavioral therapies: a call for combinatory congruency. Cogn. Behav. Pract..

[bb0260] Ong C.W., Pierce B.G., Klein K.P., Hudson C.C., Beard C., Björgvinsson T. (2022). Longitudinal measurement invariance of the PHQ-9 and GAD-7. Assessment.

[bb0270] Patel T., Brewin C.R., Wheatley J., Wells A., Fisher P., Myers S. (2007). Intrusive images and memories in major depression. Behav. Res. Ther..

[bb0275] Peper E., Lin I.-M. (2012). Increase or decrease depression: how body postures influence your energy level. Biofeedback.

[bb0280] Plessen C.Y., Panagiotopoulou O.M., Tong L., Cuijpers P., Karyotaki E. (2025). Digital mental health interventions for the treatment of depression: a multiverse meta-analysis. J. Affect. Disord..

[bb0285] Richards D., Richardson T. (2012). Computer-based psychological treatments for depression: a systematic review and meta-analysis. Clin. Psychol. Rev..

[bb0290] Rosseel Y. (2012). lavaan: an R package for structural equation modeling. J. Stat. Softw..

[bb0295] Rutherford B., Wager T., Roose S. (2010). Expectancy and the treatment of depression: a review of experimental methodology and effects on patient outcome. Curr. Psychiatr. Rev..

[bb0300] Schröder J., Berger T., Westermann S., Klein J.P., Moritz S. (2016). Internet interventions for depression: new developments. Dialogues Clin. Neurosci..

[bb0305] Simmons J.P., Simonsohn U. (2017). Power posing: P-curving the evidence. Psychol. Sci..

[bb0310] Teasdale J.D., Barnard P.J. (1993).

[bb0315] Titov N., Dear B.F., Johnston L., Lorian C., Zou J., Wootton B., Spence J., McEvoy P.M., Rapee R.M. (2013). Improving adherence and clinical outcomes in self-guided internet treatment for anxiety and depression: randomised controlled trial. PLoS One.

[bb0320] Tong A.C., Ho F.S., Chu O.H., Mak W.W. (2023). Time-dependent changes in depressive symptoms among control participants in digital-based psychological intervention studies: meta-analysis of randomized controlled trials. J. Med. Internet Res..

[bb0325] Tseng V.W.S., Tharp J.A., Reiter J.E., Ferrer W., Hong D.S., Doraiswamy P.M., Nickels S., Project Baseline Health Study Research Group (2024). Identifying a stable and generalizable factor structure of major depressive disorder across three large longitudinal cohorts. Psychiatry Res..

[bb0330] Twomey C., O'Reilly G. (2017). Effectiveness of a freely available computerised cognitive behavioural therapy programme (MoodGYM) for depression: meta-analysis. Aust. N. Z. J. Psych..

[bb0015] van Ballegooijen W., Cuijpers P., van Straten A., Karyotaki E., Andersson G., Smit J.H., Riper H. (2014). Adherence to internet-based and face-to-face cognitive behavioural therapy for depression: a meta-analysis. PLoS One.

[bb0335] VanderWeele T.J., Vansteelandt S. (2022). A statistical test to reject the structural interpretation of a latent factor model. J. R. Stat. Soc. Ser. B Stat Methodol..

[bb0340] Veenstra L., Schneider I.K., Koole S.L. (2017). Embodied mood regulation: the impact of body posture on mood recovery, negative thoughts, and mood-congruent recall. Cognit. Emot..

[bb0345] Vu L.G., Le L.K., Dam A.V.T., Nguyen S.H., Vu T.T.M., Trinh T.T.H., Do A.L., Do N.M., Le T.H., Latkin C., Ho R.C.M., Ho C.S.H. (2022). Factor structures of Patient Health Questionnaire-9 instruments in exploring depressive symptoms of suburban population. Front. Psychol..

[bb0350] Ware J.E., Sherbourne C.D. (1992). The MOS 36-item short-form health survey (SF-36). Conceptual framework and item selection. Med. Care.

[bb0355] Williams J.M.G., Barnhofer T., Crane C., Herman D., Raes F., Watkins E., Dalgleish T. (2007). Autobiographical memory specificity and emotional disorder. Psychol. Bull..

[bb0360] Wolf S., Seiffer B., Zeibig J.-M., Frei A.K., Studnitz T., Welkerling J., Meinzinger E., Bauer L.L., Baur J., Rosenstiel S., Fiedler D.V., Helmhold F., Ray A., Herzog E., Takano K., Nakagawa T., Günak M.M., Kropp S., Peters S., Flagmeier A.L., Zwanzleitner L., Sundmacher L., Ramos-Murguialday A., Hautzinger M., Sudeck G., Ehring T. (2024). A transdiagnostic group exercise intervention for mental health outpatients in Germany (ImPuls): results of a pragmatic, multisite, block-randomised, phase 3 controlled trial. Lancet Psychiatry.

[bb0365] Yardley L., Morrison L., Bradbury K., Muller I. (2015). The person-based approach to intervention development: application to digital health-related behavior change interventions. J. Med. Internet Res..

[bb0370] Zamoscik V.E., Schmidt S.N.L., Gerchen M.F., Samsouris C., Timm C., Kuehner C., Kirsch P. (2018). Respiration pattern variability and related default mode network connectivity are altered in remitted depression. Psychol. Med..

