# Supplementary material for: Comparing a body-oriented and a cognitive online intervention for adults with depressive symptoms: A three-arm randomized controlled trial
Source: Internet Interv. 2026 Oct 21;46:101016. doi: 10.1016/j.invent.2026.101016 (PMC13717113; doi:10.1016/j.invent.2026.101016)
Supplement: Supplementary file 1 [file mmc1.docx]

Table 1. Overview of the Body, Breath, and Mind (BBM) Intervention

| Module | Qi Gong exercises |  | Behavior activation |
| --- | --- | --- | --- |
| 1 | a) Shaking the body (TCM aim: removing stagnation of blood [Yue] and Qi) b) Tapping the body (TCM aim: removing stagnation of blood [Yue] and Qi) c) Qi Gong deep relaxation (TCM aim: reducing exhaustion [Xu]) |  | Psychoeduction: basic information on associations between body, activity and mood. |
| 2 | a) ‘Purification’ breathing (TCM aim: removing pathogenic energy [Xie Qi]) b) ‘Nourishing’ breathing (TCM aim: replenish excessive lowered energy level [Qi Chen]) |  | Development of an individualized model of maintaining factors of negative and depressed mood with a specific focus on activity and avoidance. |
| 3 | a) ‘Lift, open, lower, close’ in an activating style : Qi Gong movement (TCM aim: reducing excessive lowered energy level [Qi Chen]; orienting the body upwards to strengthen Yang [activity] in an activating way) b) ‘Lift, open, lower, close’ in an relaxed style : Qi Gong movement (TCM aim: reducing excessive lowered energy level [Qi Chen] ; orienting the body upwards to strengthen Yang [activity] in an relaxed way) |  | Introduction to the concept of values and clarification of the individual values |
| 4 | Intonating the Ha-sound (TCM aim: removing blockages of subtle channels of the heart by phlegm [Tan]) |  | Planning of value-based activities for the next week. |
| 5 | The inner smile: learning to smile kindly at the heart (TCM aim: removing blockages of subtle channels of the heart by phlegm [Tan]) |  | Analyzing difficulties in implementing value based activities. Planning value-based activities for the next week. |
| 6 | Deepening the inner smile: letting the inner smile expand into the surrounding and to other people (TCM aim: reducing weakness of the Heart-Mind [Shen], widening the heart and allowing the energy of the heart to radiate) |  | Introduction of mindfulness: being in non-judgmental contact with the here-and-now reduces negative thinking patterns and can support the implementation of value-based activities in the face of challenges. Mindfulness exercises. Planning value-based activities for the next week. |
| 7 | Standing like a tree: Imagine oneself turning into a tree (TCM aim: developing bodily and psychological stability through ‘grounding’; using the energy of the plants to replenish excessive lowered energy level [Qi Chen]) |  | Rumination as a hindrance in the implementation of valued-based action: Identifying rumination and developing strategies to quit rumination. Planning value-based activities for the next week. |
| 8 | 30-minute condensed exercises which consist of short versions of all exercises taught during the program. This condensed exercise could be used for regular practice after the end of the program. |  | Evaluation of the program (What elements were helpful?), finding a good reason to continue the practice and developing a plan to continue with Qi Gong practice and value-based activities. |

Table 2. Overview of the Moodgym Intervention

| Module | Content |
| --- | --- |
| Module 1: Feelings | Why you feel the way you do   a. Connections between thoughts and feelings b. Identifying the emotional consequences of negative thoughts c. Understanding WUTIWUF (what you think is what you feel) |
| Module 2: Thoughts | Changing the way we think to feel better   a) Types of dysfunctional thinking and how these might be contested b) Common problem areas such as authority and intimate relationships c) Improving self-esteem |
| Module 3: Unwarping | Changing warped thoughts   a) Different ways to change dysfunctional thinking b) Identifying personal vulnerabilities c) More help with self-esteem through actively increasing positive events |
| Module 4:  Destressing | Knowing what makes you upset   a) Identifying situations or events which might trigger stress or warped thoughts b) Relaxation methods (includes downloads) c) Problem-solving |
| Module 5: Relationships | Break-ups and how you were raised   a) How to cope with and grow from relationship break-ups b) Mum and Dad issues (or how you were raised) |
| Work-book | Easy access to quizzes and diaries   a) Retake quizzes and compare your results over time b) Access exercises and diaries from each of the modules |
